# Establishing Best Practices for Clinical GWAS: Tackling Imputation and Data Quality Challenges

**DOI:** 10.3390/ijms26136397

**Published:** 2025-07-03

**Authors:** Giorgio Casaburi, Ron McCullough, Valeria D’Argenio

**Affiliations:** 1 Department of Bioinformatics and Innovation Strategy, SOLVD Health, 1600 Faraday Ave., Carlsbad, CA 92008, USA; gcasaburi@solvdhealth.com; 2 Department of Clinical Operations, SOLVD Health, 1600 Faraday Ave., Carlsbad, CA 92008, USA; rmccullough@solvdhealth.com; 3 Department for the Promotion of Human Sciences and Quality of Life, San Raffaele Roma Open University, 00166 Rome, Italy; 4 CEINGE-Biotecnologie Avanzate Franco Salvatore, 80145 Napoli, Italy

**Keywords:** genome-wide association studies (GWASs), genotype imputation, molecular diagnostics, precision medicine

## Abstract

Genome-wide association studies (GWASs) play a central role in precision medicine, powering a range of clinical applications from pharmacogenomics to disease risk prediction. A critical component of GWASs is genotype imputation, a computational method used to infer untyped genetic variants. While imputation increases variant coverage by estimating genotypes at untyped loci, this expanded coverage can enhance the ability to detect genetic associations in some cases. However, imputation also introduces biases, particularly for rare variants and underrepresented populations, which may compromise clinical accuracy. This review examines the challenges and clinical implications of genotype imputation errors, including their impact on therapeutic decisions and predictive models, like polygenic risk scores (PRSs). In particular, the sources of imputation errors have been deeply explored, emphasizing the disparities in performance across ancestral populations and downstream effects on healthcare equity and addressing ethical considerations surrounding the access to equitable genomic resources. Based on the above, we propose evidence-based best practices for clinical GWAS implementation, including the direct genotyping of clinically actionable variants, the cross-population validation of imputation models, the transparent reporting of imputation quality metrics, and the use of ancestry-matched reference panels. As genomic data becomes increasingly adopted in healthcare systems worldwide, ensuring the accuracy and inclusivity of GWAS-derived insights is paramount. Here, we suggest a framework for the responsible clinical integration of imputed genetic data, paving the way for more reliable and equitable personalized medicine.

## 1. Introduction

The incorporation of genomic information into clinical care is reshaping healthcare, driven by the reduced sequencing costs, advances in precision medicine, and widespread availability of genomic datasets [1]. Genome-wide association studies (GWASs) have been instrumental in identifying genetic variants linked to a variety of complex diseases and traits. These findings have been valuable for predicting disease risk, personalizing treatment plans, and enhancing patient care. In clinical settings, GWAS findings have been employed extensively in pharmacogenomics to predict drug responses [2], in risk prediction for diseases like cardiovascular disease and type 2 diabetes [3], and to guide cancer screening and prevention strategies [4]. More recently, GWASs have been leveraged to explore genetic predispositions to complex, multifactorial conditions such as opioid use disorder [5]. These efforts have identified the risk loci associated with opioid sensitivity, addiction vulnerability, and the response to treatment, laying the groundwork for more personalized strategies in prevention, early intervention, and therapeutics [6,7]. Despite the huge amount of data produced to date and the increasing number of clinical applications, the clinical utility of the GWAS findings hinges on the quality and accuracy of the underlying genomic data. This particularly applies to genotype imputation, which plays a pivotal role in expanding the variants’ coverage and guiding the downstream analyses. Indeed, imputation errors can compromise downstream analyses, distort risk classification, and, ultimately, misguide clinical decision-making. Classical GWAS-based strategies have been centered on identifying individual single-nucleotide polymorphisms (SNPs) associated with traits, estimating each SNP’s effect size (r^2^) in isolation [8]. While this approach has yielded important insights, it accounts for only a small portion of the heritability underlying complex traits. This limitation has driven the emergence of polygenic risk scores (PRSs), which aggregate the effects of numerous genetic variants into a single composite score [9]. Therefore, by capturing the cumulative influence of multiple loci, including many variants with individually small effects that do not reach genome-wide significance, PRSs offer a more comprehensive estimate of genetic liability. This aggregation allows PRSs to explain a greater proportion of phenotypic variance, thereby helping to address the missing heritability observed when only considering the genome-wide significant loci [10]. Originally introduced in psychiatric genomics [11], PRSs are now widely used across diverse disease contexts, reinforcing the understanding that the clinical phenotype of most common diseases is influenced by hundreds or even thousands of variants [11]. The aggregation of small-effect variants in PRSs enables the stratification of individuals across a continuum of genetic risk, helping to identify those at the highest and lowest ends who may benefit from targeted screening or preventive interventions [12,13]. Importantly, this approach also helps to address the well-known ‘missing heritability’ problem, the discrepancy between heritability estimates from family studies and the smaller proportion accounted for by GWAS-identified variants [11,14]. In practice, a person’s genome is scanned for known risk alleles, each weighted by its estimated effect size, and these values are summed to produce an overall PRS [13,15].

Despite the growing promise of PRSs, their accuracy and clinical relevance remain tightly linked to the quality of the underlying GWAS data. As these findings begin to impact real-world clinical decisions, it becomes increasingly important to address the underlying limitations, particularly those introduced during genotype imputation. Errors at this stage, coupled with the biases in reference panels and interpretive uncertainty, can compromise genomic validity and risk widening existing health disparities.

To address these challenges, this review focuses on the pivotal role of genotype imputation in GWASs, examining the strengths, limitations, and implications for clinical applications. We detail how the imputation quality influences downstream analyses, especially polygenic risk modeling, and highlight the disparities in performance across ancestries. Finally, we propose a set of best practices designed to improve the imputation accuracy and promote the equitable, reliable integration of GWAS findings into patient care.

## 2. Genotype Imputation in GWASs

Genotype imputation is a computational method used to predict genetic variants that are not directly genotyped, often referred to as untyped variants. In large-scale studies, genotyping every SNP is often impractical due to the cost and array limitations. Instead, researchers typically genotype a subset of common variants and rely on imputation to estimate the remaining genotypes based on a reference panel [16].

**Figure 1:**
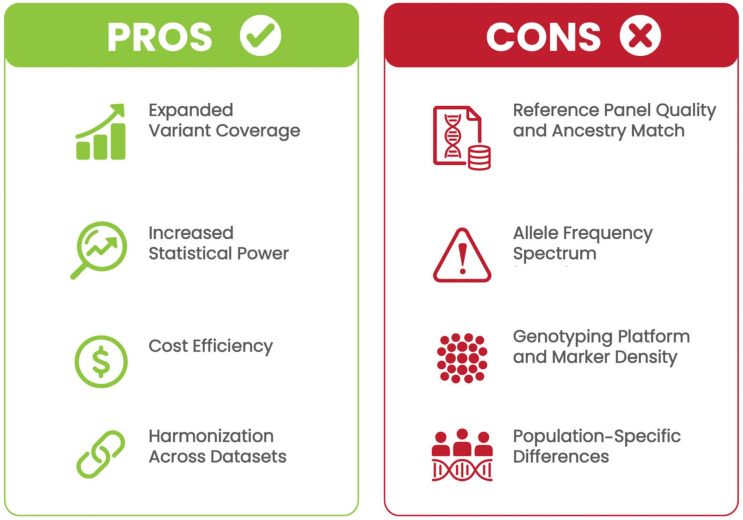
Pros and cons of genotype imputation in clinical GWAS applications. Benefits include expanded variant coverage, improved statistical power, cost efficiency, and harmonization across datasets. Limitations include dependency on reference panel quality, allele frequency spectrum biases, genotyping platform variability, and population-specific imputation challenges.

Genotype imputation offers several key advantages (Figure 1).

First, imputation enables the detection of genetic variants not directly captured by genotyping arrays, increasing the comprehensiveness of genetic analyses. This is particularly valuable for capturing low-frequency and rare variants that are typically underrepresented on genotyping arrays, as these variants can have significant effects on disease risk [17]. By expanding the number of SNPs tested, imputation also improves the power to detect associations, particularly for complex diseases with small-effect variants spread across the genome [18]. Moreover, rather than conducting whole-genome sequencing, researchers can genotype a limited set of markers and use imputation to infer the rest, significantly reducing costs while maintaining analytical depth [19]. Finally, imputation facilitates the combination of datasets from different cohorts and genotyping platforms. When the variant overlap is limited across arrays, imputation fills in the missing data, enabling meta-analyses and multi-cohort studies. For example, successive generations of Affymetrix or Illumina genotyping arrays may differ in their SNP content; imputation facilitates harmonization by inferring a common set of variants across platforms [20]. Together, these advantages have made genotype imputation a standard component of modern GWAS workflows. However, the effectiveness of imputation depends not only on the design of the genotyping array or the traits under study, but also on the quality and structure of the reference data used to infer missing variants. In fact, imputation heavily relies on reference panels (i.e., large datasets containing phased haplotypes that serve as genomic templates). These reference panels allow researchers to statistically estimate the most likely genotypes at untyped loci by comparing the observed haplotype patterns in the study population [21].

The imputation process typically involves two key steps, the phasing and imputation of the missing genotypes. Phasing determines which alleles are inherited together on the same chromosome (i.e., from the same parent). This step reconstructs an individual’s haplotypes by analyzing the patterns of linkage disequilibrium (LD), the non-random association of alleles at different genetic loci in the observed genotype data (e.g., certain variants are inherited together more frequently than expected by chance). Accurate phasing is critical for successful imputation, as it establishes the framework for comparing haplotypes to those in the reference panel [22]. After phasing, the genotypes are compared against the reference panel and statistical models use the shared haplotype structure to infer the most probable allele(s) at untyped loci [23]. For example, if an individual’s genotype is missing at a particular SNP, the surrounding markers and known LD patterns in the reference panel are used to predict the most likely genotype. This process enables researchers to impute millions of additional variants not directly genotyped, including many low-frequency and rare-variant alleles. As mentioned above, the accuracy of these imputations depends heavily on the quality of the reference panel and the ancestral similarity between the reference and the study population. However, when performed appropriately, imputation greatly increases the resolution of GWASs and improves the discovery of trait-associated loci.

### 2.1. Imputation Methods

A wide range of genotype imputation algorithms have been developed, each offering distinct advantages and limitations. These tools vary in computational efficiency, accuracy across different variant frequencies, and suitability for specific population structures or dataset scales. Importantly, no single method consistently outperforms others across all scenarios. Thus, the choice of the imputation tool should be guided by the study’s objectives, the available computational resources, the allele frequency spectrum of interest, and the genetic diversity of the target population. Table 1 summarizes several widely used imputation algorithms, outlining their key strengths, limitations, and recommended contexts for application in genomic research and clinical settings.

#### Potential and Limitations of Deep-Learning-Based Imputation

Recent advances in deep learning have led to the development of algorithms such as DeepImpute, which apply neural networks to model the complex relationships among genetic variants and improve imputation accuracy, particularly for rare variants. These methods can capture non-linear dependencies in genomic data beyond what traditional LD-based methods achieve. However, deep-learning approaches come with notable limitations. First, they require extensive, high-quality training datasets representative of the target ancestries to achieve accurate predictions. This poses a challenge for underrepresented groups, where large-scale genomic data are often lacking, leading to biased or inaccurate imputations. Additionally, deep-learning models typically involve increased computational complexity and longer training times compared to traditional methods like Minimac4 [26] or Beagle [25], which can limit their feasibility in large-scale or resource-constrained settings. Moreover, the “black box” nature of deep-learning models can complicate the interpretation of imputation results and hinder clinical adoption, where transparency and explainability are essential. Despite these challenges, as diverse genomic datasets continue to grow globally, deep-learning-based imputation methods hold promise for improving accuracy, especially for rare variants and poorly covered genomic regions.

### 2.2. Quality Metrics for Imputation

The accurate evaluation of imputation results is essential for ensuring the reliability of downstream analyses, particularly in clinical applications where variants’ misclassification may have significant implications in patients’ diagnostic process and consequent clinical management. Several key metrics are commonly used to assess the imputation quality, including the following:

1. INFO Score: The INFO score estimates the squared correlation between imputed and true genotypes, ranging from 0 to 1. Higher values indicate greater confidence. A threshold of INFO > 0.8 is typically used for inclusion in downstream analyses, although lower thresholds (e.g., 0.6–0.7) may be acceptable when evaluating low-frequency variants or when using well-matched reference panels. However, it is important to recognize that a fixed INFO threshold may not be universally appropriate across all populations. For example, in ancestrally diverse or admixed cohorts, such as African Americans, greater haplotype diversity and shorter linkage disequilibrium blocks can reduce imputation accuracy, especially for rare variants. In these contexts, more stringent thresholds (e.g., INFO > 0.9) may be considered for variants with a high clinical impact to minimize false positives, while slightly relaxed thresholds might be acceptable for exploratory analyses or polygenic risk score construction when maximizing variant inclusion is important. Threshold selection should be guided by the imputation performance of the reference panel in the target population, variant allele frequency, and the intended application, and should be transparently reported with justification. INFO scores are also influenced by the minor allele frequency (MAF); rare variants tend to have lower INFO scores due to the greater uncertainty (Table 2) [29].
2. Imputation R-squared (r^2^): This metric also estimates the correlation between imputed and actual genotypes, expressing the proportion of variance explained. It is especially useful in regression-based analyses, and, while INFO and r^2^ are often used interchangeably, they are computed differently across platforms (e.g., IMPUTE vs Minimac) and may not be directly comparable (Table 2) [30].
3. Minor Allele Frequency (MAF): Variants with a low MAF (e.g., <0.01) are more difficult to impute accurately and often yield lower confidence scores. In clinical contexts, these variants require more stringent quality control to avoid spurious associations or false positives. For example, a rare variant associated with drug metabolism, such as DPYD rs3918290, which influences fluoropyrimidine toxicity, has an MAF below 1% in most populations. If poorly imputed, this variant could lead to incorrect dosing recommendations in pharmacogenomic settings. Ensuring high-quality imputation or confirming such variants with direct genotyping is critical when the clinical consequences are significant (Table 2) [31].
4. Hard-Call Concordance: This metric assesses the agreement between the imputed genotypes and directly genotyped variants in a reference subset of samples. It provides a direct measure of imputation accuracy but requires access to a gold-standard dataset for comparison (Table 2) [32].

When interpreting imputed genotypes in clinical settings, these metrics should guide both variants’ filtering and confidence reporting. Filtering variants with INFO or r^2^ < 0.8 is a standard practice to reduce the risk of imputation errors. Additionally, visual inspection (e.g., using regional association plots or imputation quality plots) is recommended to identify outliers, artifacts, or unexpected clustering patterns. For example, variants with high minor allele frequencies (MAFs) but disproportionately low INFO scores (e.g., INFO < 0.3 when similar-frequency variants have INFO > 0.8) can indicate problematic regions or technical artifacts. Likewise, abrupt changes in the distribution of INFO scores across loci or samples may suggest reference panel mismatches or genotyping errors. These patterns should prompt careful review or confirmation with direct genotyping before clinical interpretation.

### 2.3. Factors Affecting Imputation Accuracy

Although imputation quality can be quantified using metrics such as the INFO score or imputation r^2^, the accuracy of these values is fundamentally shaped by the upstream design and study-specific variables. In clinical genomics, understanding these factors is essential to minimize errors propagation and ensure the reliable interpretation of genetic findings. Factors affecting imputation accuracy include the ancestry match of the reference panel, allele frequency spectrum, genotyping array resolution, and platform heterogeneity (Figure 1).

Indeed, the imputation accuracy is strongly influenced by the degree of ancestral concordance between the study cohort and the reference panel. When the genetic ancestry of the study population closely aligns with that of the reference panel, the imputation performance (particularly for rare variants) is significantly enhanced. Conversely, mismatches in ancestry can lead to inaccurate imputations due to differences in the LD structure and allele frequency distributions. For instance, applying a European-derived reference panel to an African American cohort often yields reduced accuracy, as the LD patterns and ancestry-specific variants may not be adequately captured. To address this, researchers should prioritize the use of ancestry-matched or multi-ethnic reference panels, such as those derived from the 1000 Genomes Project or Haplotype Reference Consortium, to maximize the imputation fidelity across diverse populations [30].

Moreover, the imputation accuracy is generally higher for common variants (MAF > 5%) than for rare ones (MAF < 1%). In fact, rare variants tend to yield lower confidence scores and higher error rates, introducing potential bias into the downstream analyses, such as association testing or PRS construction [33]. These variants are often population-specific and underrepresented in most reference panels, which limits the ability of imputation algorithms to accurately infer their genotypes. Despite their low frequency, rare variants can have significant clinical relevance, especially in Mendelian disorders or complex diseases influenced by founder effects [34]. For this reason, it is essential that we rigorously assess the imputation quality of rare variants before incorporating them into clinical interpretations or predictive models.

The density of genotyping arrays also significantly impacts imputation resolution. High-density arrays, such as the Illumina OmniExpress (~700,000 SNPs), better capture LD patterns, improving the imputation accuracy. However, these arrays come with higher costs, necessitating trade-offs based on research or clinical objectives. An inadequate marker density can compromise downstream analyses, such as disease risk prediction or pharmacogenomic interpretation [35]. To optimize the imputation performance, genotyping arrays often include tag SNPs (i.e., variants in strong LD with other loci) to efficiently represent a broader genomic variation. Emerging imputation-aware design strategies further enhance marker selection by tailoring SNP inclusion to specific populations or genomic regions, thereby improving overall efficiency [36]. Given the implications for clinical decision-making, researchers must carefully balance the platform cost, marker density, and imputation quality during study design. Rigorous quality control is essential in order to mitigate errors stemming from poor marker coverage or platform heterogeneity.

Finally, the genotype imputation performance is not uniform across populations. It is generally highest in individuals of European ancestry, who are overrepresented in most existing reference panels [37]. In contrast, individuals from underrepresented populations, including those of African, Hispanic/Latino, Indigenous, or admixed backgrounds, often experience a lower imputation accuracy due to the distinct patterns of LD and allele frequency distributions [33]. These discrepancies can introduce systematic bias in downstream analyses. For example, PRSs derived from European-based GWASs often fail to transfer accurately to non-European populations, resulting in risk misclassification and reduced clinical utility [13]. Without appropriate adjustments, this disparity risks exacerbating existing healthcare inequities by offering more reliable genomic predictions for some populations than others. Several strategies can mitigate these challenges. Ancestry-informed calibration methods, such as principal component analysis (PCA) and local ancestry modeling, can help account for the population structure and improve the interpretability of PRSs across diverse cohorts [38]. Additionally, the use of ancestry-specific or multi-ethnic reference panels, as well as emerging cross-ancestry imputation frameworks, can improve imputation fidelity and reduce bias in genomic prediction [39]. Ensuring equitable genomic care requires continued investments in globally representative sequencing efforts and a commitment to building inclusive reference datasets. As summarized in Table 3, the imputation accuracy varies considerably by ancestry group and allele frequency. European populations typically show higher r^2^ values for both common and rare variants, while non-European populations, particularly underrepresented or admixed groups, demonstrate reduced accuracy, especially for rare alleles. These disparities are influenced by reference panel representation and highlight the need for population-specific imputation resources.

These results illustrate the persistent disparities in imputation quality across global populations, highlighting the urgent need for more inclusive reference panels and the context-specific validation of imputed genotypes in clinical workflows.

## 3. GWAS Clinical Actionability, Genotyping Confirmation, and Transparent Reporting

Since its development about 20 years ago, GWASs have largely been used to improve our understanding of human diseases and to develop novel diagnostic and/or therapeutic strategies [44]. The increasing availability of genomic data, and their linked phenotypes, has further prompted the diffusion of GWAS applications in clinical settings [45]. In this context, the correct downstream interpretation of genomic data is crucial.

In particular, genotype imputation plays an increasingly central role in clinical genomics, with applications ranging from pharmacogenomics and disease risk prediction to identifying high-risk individuals for population screening. However, its utility in clinical decision-making must be tempered by the recognition that imputed genotypes, especially those involving low-frequency variants or underrepresented populations, may carry uncertainty that impacts patient care. For example, variants in the *OPRM1* gene have been robustly associated with opioid use disorder (OUD). These variants are critical for understanding opioid receptor function and addiction risk. However, the imputation accuracy for such variants can be limited in underrepresented populations due to ancestry-specific reference panel deficiencies, which may lead to misimputation and inappropriate treatment strategies [46].

For this reason, direct genotyping remains the preferred approach to analyze other clinically significant variants used in pharmacogenomic guidance (e.g., *CYP2C19* for clopidogrel metabolism) [47], or for disease risk classification (e.g., *BRCA1/2*, and *LDLR*) [48]. Indeed, the misimputation of these actionable variants can result in inappropriate drug selection, suboptimal dosing, or missed diagnoses. In the context of OUD treatment, where genetic information informs medication strategies, such as methadone or buprenorphine dosing, orthogonal validation, through methods like Sanger sequencing or PCR-based assays, is essential in order to confirm the imputed results prior to clinical implementation. Furthermore, the inherent limitations of genotype imputation in inaccessible regions of the genome exacerbate these challenges. Studies have shown that genotyping chips and imputation reference panels are systematically biased against inaccessible genomic regions, leading to missed variants and spurious associations [49]. This highlights the need for the careful interpretation of the imputed data and emphasizes the importance of direct genotyping or high coverage sequencing when dealing with clinically actionable variants.

Transparent reporting is equally critical. Imputed variants should be clearly flagged in clinical summaries, with confidence metrics, such as INFO or r^2^ scores, provided. Disclaimers should accompany the results that may be less reliable due to ancestry mismatch or low minor allele frequency. This enables clinicians and laboratories to assess the reliability of the imputed findings and consider confirmatory testing when needed.

Key clinical applications that may be impacted by imputation will be briefly discussed below.

### 3.1. Drug Dosing and Pharmacogenomics

GWASs have largely been used to carry out pharmacogenomic studies and clarify the mechanisms underlying individuals’ response to specific therapies [50]. To date, several studies have been performed, highlighting the significant association between specific SNPs and drugs’ efficacy and/or adverse reactions [50]. In this context, very recent studies have assessed the reliability of GWASs in identifying SNPs related to drug resistance in focal epilepsy [51] to myelosuppression in non-small-cell lung cancer patients treated with platinum-based chemotherapy [52] and detecting genetic variants influencing the response to vitamin D3 supplementation in atherosclerotic patients [53].

In all these cases, the correct interpretation of the obtained genomic data may be challenging, affecting the biological implications of the detected associations. For drugs with narrow therapeutic indices, like warfarin or clopidogrel, the misimputation of pharmacogenetic variants may lead to adverse outcomes. The accurate identification of pharmacogenomic alleles is critical for optimizing drug efficacy and minimizing risks, especially in anticoagulant therapy where genetic variation significantly impacts the dosing requirements. For instance, variants in *CYP2C9* and *VKORC1* are well-known predictors of warfarin metabolism and sensitivity, with minor alleles like CYP2C9 *2 and *3 linked to reduced drug metabolism [54]. Structural variants and copy number variations (CNVs) present additional challenges in pharmacogenomics. These variants are poorly captured through imputation and require alternative detection methods, such as comparative genomic hybridization (CGH) arrays or whole-genome sequencing (WGS), for accurate identification [55]. CNVs can significantly affect the drug response by altering the gene dosage or regulatory elements, making their reliable detection essential for assessing drug efficacy and safety. This is particularly relevant in drug response assessment, where undetected structural variations may lead to suboptimal treatment outcomes or an increased risk of adverse events [56]. In clinical practice, the limitations of imputation for structural variants highlight the need for direct genotyping or high-resolution sequencing platforms to ensure accurate pharmacogenomic profiling. This approach not only improves therapeutic precision but also mitigates the risks associated with misimputation in critical drug–response pathways.

### 3.2. Polygenic Risk Prediction

PRSs represent an emerging tool for deciphering the complexity of human diseases. As a result of the availability of genomic databases, PRSs have been developed to study the molecular bases of an increasing number of complex diseases, paving the way to precision medicine. Indeed, PRSs can estimate diseases’ risk by supporting the correct diagnosis and therapeutic choices [57].

Imputation expands SNP coverage, improving PRS modeling for complex diseases like cardiovascular disease, cancer, and diabetes [58,59,60]. This enhanced variant density enables the more precise stratification of individuals based on their genetic risk profiles, supporting tailored prevention strategies. Transparent reporting is crucial in this domain. Imputed variants used in PRS calculations should be flagged with confidence metrics, such as INFO or r2 scores, to indicate the reliability of the imputation process. Disclaimers should accompany the results that may be less reliable due to factors such as ancestry mismatch or low minor allele frequency [61]. For example, studies have shown that the imputation accuracy decreases significantly for low-frequency variants (minor allele frequency < 5%), and errors in these regions can undermine PRS validity. This is particularly relevant for diseases where rare variants play a significant role in genetic risk. Enhanced variant density through imputation has demonstrated utility in various contexts. For instance, the PRS models for coronary artery disease integrate thousands of genetic loci associated with lipid metabolism and inflammatory pathways, while breast cancer PRS models incorporate imputed variants linked to hormonal regulation and DNA repair mechanisms. In diabetes, imputed variants associated with insulin secretion and glucose homeostasis improve the early identification of high-risk individuals who may benefit from lifestyle interventions or enhanced monitoring [61]. Because the reliability of PRS models depends heavily on imputation quality and the representativeness of the reference panels used, transparent reporting in clinical summaries is crucial. Clear documentation enables clinicians and diagnostic laboratories to assess the confidence of the imputed findings and determine whether confirmatory testing is warranted. As PRSs gain traction in clinical practice, maintaining transparency around imputation metrics will be critical to upholding scientific rigor and maximizing their impact on precision medicine.

### 3.3. Identification of High-Risk Individuals

Even in the absence of a strong family history, imputed data can uncover genetic risk, identifying patients who may benefit from early intervention, lifestyle modification, or enhanced screening (e.g., in breast cancer or coronary artery disease). For example, imputed variants associated with breast cancer risk (e.g., *BRCA1/2*) or coronary artery disease (e.g., *LDLR*) can reveal a disease susceptibility that might otherwise remain undetected. This capability is particularly valuable in clinical settings where early detection and preventive measures can significantly improve outcomes. For instance, it has been reported that imputation accuracy declines in populations underrepresented in reference panels, potentially leading to false negatives or false positives in the high-risk group [62]. By clearly documenting these limitations, clinicians and laboratories can better assess whether confirmatory testing, such as direct genotyping or sequencing, is warranted. To fully realize the potential of genomic medicine, the responsible application of imputed data must prioritize accuracy, transparency, and inclusivity. Transparent reporting, through the clear documentation of imputation metrics, ancestry-specific limitations, and variant reliability, empowers clinicians and researchers to make informed decisions about patient care and study design.

**Figure 2:**
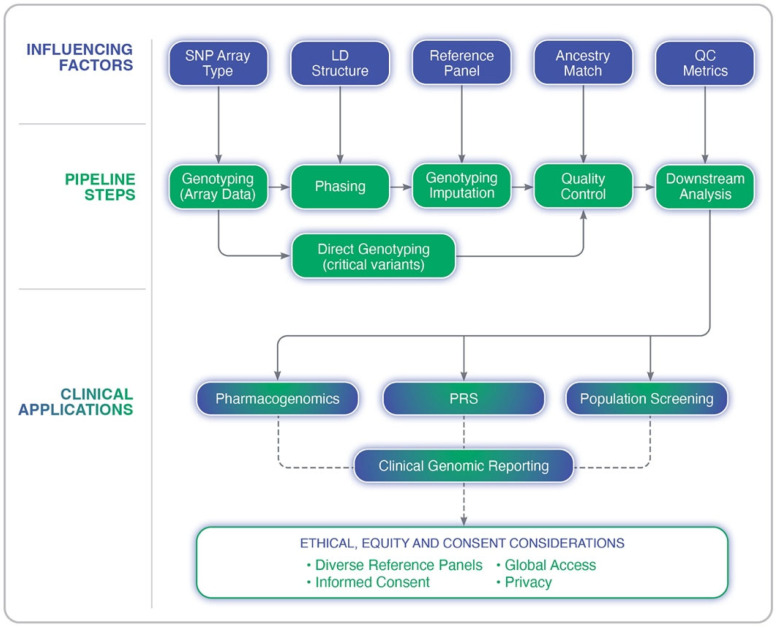
Overview of the genotype imputation pipeline in genome-wide association studies. The diagram illustrates key analytical steps (e.g., genotyping, phasing, imputation, and quality control), upstream influencing factors (e.g., LD structure, SNP array type, and reference panel quality), and clinical applications such as pharmacogenomics, polygenic risk scoring (PRS), and population screening. Ethical and equity considerations are also highlighted, including the importance of diverse reference panels, informed consent, and privacy.

Many of the challenges discussed here, including ancestry representation, quality control measures, and the validation of findings, are all central to GWAS design and interpretation. The following section provides a detailed framework for conducting GWASs with scientific rigor while ensuring equitable representation across diverse populations. The genotype imputation process and its clinical implications is outlined in Figure 2, depicting the key steps in the imputation pipeline, the factors that influence its accuracy, and the downstream applications in precision medicine.

## 4. Best Practices for GWAS Implementation in Clinical Settings

**Figure 3:**
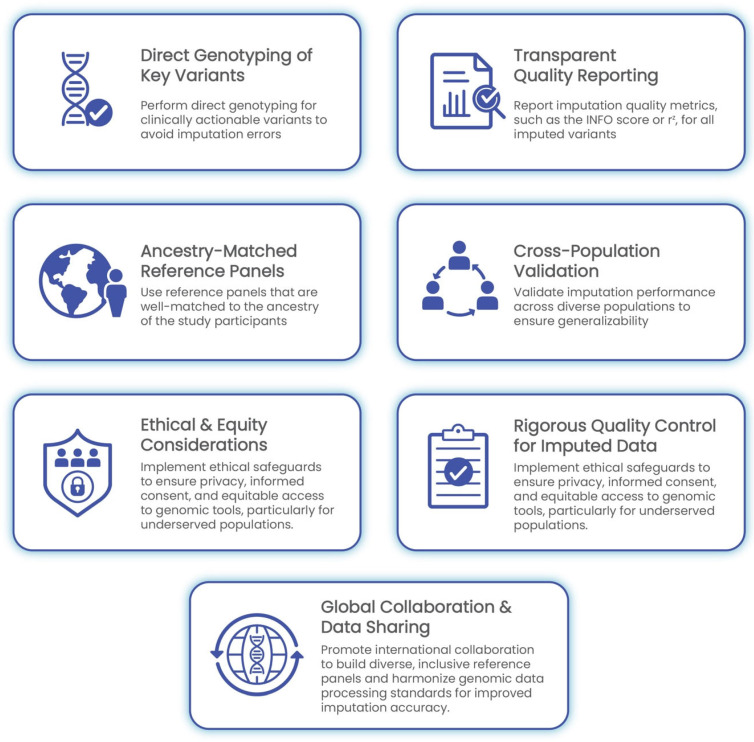
Best practices for clinical implementation of GWASs and genotype imputation. This framework outlines seven core recommendations: direct genotyping of clinically actionable variants, transparent reporting of imputation quality metrics, cross-population validation, ancestry-matched reference panels, rigorous quality control, ethical and equity safeguards, and global collaboration. Together, these practices promote accurate, inclusive, and responsible integration of genomic data into healthcare.

The implementation of GWASs in clinical settings demands meticulous attention to data quality, methodological rigor, and population sensitivity. As outlined in the preceding sections, challenges such as genotype imputation errors, population-specific biases, and quality control gaps can significantly undermine the reliability of GWAS-derived insights. The following best practices are proposed to ensure the accurate, ethical, and equitable integration of imputed genomic data into clinical decision-making (Figure 3).

### 4.1. Rigorous Quality Control for Imputed Data

Implementing stringent quality control (QC) procedures is essential for minimizing false positives and enhancing the reliability of clinical interpretations. Widely used tools, such as PLINK [63], QCTOOL [64], and GenomeStudio (Illumina Inc.), support scalable QC workflows for GWAS data. Recommended filters include the following:

1. INFO/r^2^ thresholds: Exclude variants with INFO or r^2^ scores below a certain threshold. While a cutoff of 0.8 is commonly used, this should be adjusted based on the study goals and variant characteristics. For example, studies focusing on rare variants or underrepresented populations may benefit from more stringent thresholds to improve the reliability of imputed calls [65].
2. Minor allele frequency (MAF): Exclude variants with MAF < 0.01 unless directly genotyped. This step helps to reduce spurious associations driven by rare variants with unreliable imputation.
3. Hardy–Weinberg equilibrium (HWE): Filter variants deviating from HWE (e.g., *p* < 1 × 10^−6^). Deviations from HWE can indicate genotyping errors or ancestry stratification issues.
4. Ancestry stratification: Adjust for the genetic ancestry structure using a principal component analysis (PCA), and more advanced methods such as mixed linear models (e.g., EMMAX [66] and GEMMA [67]) or local ancestry deconvolution [68]. These methods account for subtle population structure effects that can confound association analyses. PCA captures broad-scale ancestry differences, while mixed models and local ancestry deconvolution can address more complex patterns of relatedness and admixture.
5. Batch effects: Users should test for systematic, non-biological errors using visualization techniques such as PCA and multidimensional scaling (MDS). Correct for these effects using statistical methods like linear regression, and an analysis of variance (ANOVA), or more sophisticated approaches such as ComBat [69] and mixed-effects models [70], which account for both fixed and random effects.

Emerging quality control (QC) tools, such as GWASinspector, automate the QC process by providing comprehensive metrics for large datasets, enhancing efficiency and accuracy [71]. Additionally, some pipelines integrate machine-learning (ML) techniques to identify and correct errors more effectively. For instance, tools like DeepVariant utilize convolutional neural networks (CNNs) to improve variant calling accuracy by formulating error correction as a multiclass classification problem [72]. Other studies have shown that ML-assisted classifiers can filter erroneous next-generation sequencing (NGS) reads, distinguishing true genetic variations from artifacts. These ML approaches are transforming the error correction in genomics by automating processes and improving reliability [72].

Documenting all QC procedures with version control ensures transparency and reproducibility. For example, “[Variants with INFO < 0.8, MAF < 0.01, or HWE *p* < 1 × 10^−6^ were excluded from clinical analyses. Batch effects were assessed using PCA, and linear regression was used to adjust for significant batch variables]”. These practices safeguard the integrity of genomic data and reduce the risk of misinterpretation in clinical settings, preventing false positives or inaccurate risk scores.

### 4.2. Direct Genotyping of Critical Variants: Minimizing Reliance on Imputation

For variants with a high clinical impact, such as those guiding pharmacogenomic decisions, informing diagnostic assessments, or risk classification, direct genotyping remains the gold standard, since it minimizes the risk of diagnostic errors associated with imputation.

Clinically actionable variants should be established, guided by the following prioritization criteria:

1. Established clinical guidelines: Prioritize variants included in evidence-based clinical guidelines from leading organizations such as the Clinical Pharmacogenetics Implementation Consortium (CPIC; https://cpicpgx.org/guidelines/, accessed on 5 May 2025) or the National Comprehensive Cancer Network (NCCN, https://www.nccn.org/guidelines, accessed on 5 May 2025), or relevant specialty-specific societies. These guidelines represent a consensus on variants with established clinical utility.
2. Significant effect size: Focus on variants exhibiting large effect sizes on clinically relevant phenotypes, particularly those that influence therapeutic response, disease susceptibility, or prognostic outcomes. Effect size should be considered in the context of the specific clinical application and target population [73].
3. Population-specific allele frequency: Account for the frequency of the variant in the target population. While high-frequency variants are generally prioritized, consider also including low-frequency variants that are enriched in specific populations and have substantial clinical implications within those groups [74].
4. Evidence of imputation inaccuracy: If certain variants consistently exhibit a poor imputation performance (e.g., low INFO scores, and high discordance rates) in specific populations or genomic regions, prioritize direct genotyping to ensure accurate assessment [18].

For example, *CYP2C19* variants used to guide clopidogrel therapy, *DPYD* variants influencing fluoropyrimidine metabolism, and *HLA* alleles relevant to drug hypersensitivity should be directly genotyped rather than imputed due to their established clinical actionability [75]. Genotyping methods may include real-time PCR assays (e.g., TaqMan), targeted array platforms (e.g., MassARRAY), or custom next-generation sequencing (NGS) panels. The choice of method should consider factors such as the cost, throughput, accuracy, and the number of variants being interrogated.

To ensure ongoing technical accuracy, implementing a tiered validation strategy is recommended:

1. Tier 1—Initial validation: Genotype all critical variants in a subset of samples using an orthogonal method to confirm assay accuracy and identify potential technical artifacts.
2. Tier 2—Periodic monitoring: Regularly monitor the genotyping performance using quality control metrics (e.g., call rates and allele frequencies) and implement periodic orthogonal validation in a representative subset of samples to detect any drift in assay performance or emerging technical issues.

The depth of validation should reflect the clinical consequences of misclassification. For high-stakes variants with immediate therapeutic implications, full genotyping may be warranted regardless of cost. Users should embrace modern technologies such as droplet digital PCR (ddPCR) for the precise quantification and detection of low-frequency alleles. ddPCR offers unparalleled sensitivity and precision, enabling the reliable detection of rare variants even at allele frequencies as low as 0.1%, which is critical for clinical scenarios where accurate detection can directly influence therapeutic decisions. Its ability to partition samples into tens of thousands of droplets allows for absolute quantification without the reliance on standard curves, significantly reducing error rates and increasing reproducibility. This makes ddPCR particularly valuable in detecting mutations in challenging contexts, such as circulating tumor DNA or low-prevalence alleles in liquid biopsies [76].

### 4.3. Cross-Population Validation of Imputation Models

Before deploying imputation-based tools in clinical settings, it is essential that we conduct validation studies across diverse ancestral populations. This is crucial because the imputation accuracy can vary widely depending on population-specific linkage disequilibrium (LD) patterns, allele frequencies, and reference panel representation. Studies have demonstrated substantial variations in LD patterns among populations, with African populations generally exhibiting a lower LD compared to East Asian and European populations, which have longer LD blocks due to population history and genetic drift [77]. Additionally, the representation of diverse populations in reference panels significantly impacts the imputation performance. For example, panels like TOPMed [78] and GenomeAsia have an improved accuracy for underrepresented populations by including ancestrally matched samples [79].

Multi-ethnic validation cohorts should be carefully assembled, ensuring sufficient statistical power within each ancestry group to minimize the risk of false positive and false negative results. To address this issue, first power calculations must be evaluated to determine the minimum sample size required within each ancestry group to detect statistically significant differences in imputation performance. Factors, such as the expected imputation accuracy, allele frequency of the variants of interest, and desired statistical power, have to be taken into account at this step. Subsequently, targeted recruitment strategies should be employed to ensure the adequate representation of diverse ancestral backgrounds. Collaborations with community organizations, healthcare providers serving diverse populations, and research networks focused on underrepresented groups may be an asset at this step. Genomic ancestry markers need to be employed to confirm self-reported ancestry and identify potential ancestry stratification within validation cohorts. Tools, such as principal component analysis (PCA) or ancestry deconvolution methods, can be used to assess genetic ancestry. The recruitment should strive for a balanced representation across ancestry groups to avoid bias in validation metrics. If imbalances are unavoidable, weighting strategies or stratified analyses to account for differences in sample size should be considered. Next, validation metrics have to be used to compare, within the enrolled cohorts, imputed genotypes against directly genotyped or sequenced data, stratified by ancestry. Table 4 lists the validation metrics to be employed at this step.

Moreover, population-specific discrepancies in imputation accuracy using the following strategies can be used to this aim, including the following: (i) Ancestry-Matched Reference Panels, that utilize ancestry-matched reference panels (e.g., specific 1000 Genomes or HRC subsets, and TOPMed) to improve imputation accuracy within each population; and (ii) Population-Specific Fine-Tuning, that adjust imputation algorithms and parameters to optimize performance within specific populations, accounting for differences in linkage disequilibrium patterns and allele frequencies. For variants with a consistently low imputation accuracy in specific populations, prioritize direct genotyping to ensure a reliable assessment.

Finally, longitudinal re-validation is also essential, as reference panels and algorithms evolve. This ensures the continued accuracy and reliability of imputation-based tools in clinical settings.

### 4.4. Transparent Reporting of Imputation Quality in Clinical Reports

Clinical genomic reports must clearly indicate which variants were imputed and provide quality metrics associated with each imputed variant, ensuring clinicians are well-informed about the origin and reliability of the genomic data used for decision-making. To facilitate this, reports should carry out the following:

1. Flag imputed variants: Clearly identify imputed variants using a consistent notation (e.g., asterisk, color code, or designated field) to distinguish them from directly genotyped variants.
2. Include quality scores: Provide relevant quality metrics for each imputed variant, such as INFO scores, r^2^ values, and MAF in the reference population. These metrics are essential in order to increase the confidence and reliability of the imputed calls.
3. Offer plain-language interpretations: Provide clear, concise, and context-specific interpretations of the quality metrics, enabling clinicians to understand their implications for clinical decision-making.

Table 5 below illustrates the best practices for reporting imputed variants in clinical genomics. It includes examples of pharmacogenetically actionable variants commonly used in precision medicine, their imputed genotypes, associated confidence metrics, recommended clinical actions, and guidance for confirmatory testing where appropriate.

Reports should include clear disclaimers regarding known imputation limitations in specific populations, regions of the genome, or for rare variants. Offer guidance for confirmatory testing when the imputation quality is low or when clinical decisions are high-stakes. Emphasize that confirmatory testing is particularly important in populations underrepresented in reference panels. Moreover, clinician education is critical for the appropriate interpretation and application of genomic information. Education efforts should focus on the following: (i) Workshops and Webinars, including conducting regular training sessions on imputation interpretation, quality metrics, and clinical implications; (ii) Interpretation Guides, such as developing and disseminate user-friendly guides that provide clear explanations of key concepts and metrics; and (iii) Genetics Professionals, who facilitated access, including genetic counselors and clinical geneticists for support and consultation. Following these best practices, we will ensure that clinical laboratories deliver genomic reports that are transparent, informative, and actionable.

### 4.5. Ethical and Equity Considerations

Technical advancements in genomic research must be matched with robust ethical safeguards to ensure responsible implementation. This is especially critical for historically underserved populations who may face barriers to participation and equitable access to genomic medicine. Key considerations include data privacy, culturally appropriate consent, and equitable access to genomic tools.

Indeed, the sensitive nature of genomic data necessitates comprehensive privacy measures to protect individuals from potential misuse or reidentification risks. To achieve this, users should consider that genomic data should be encrypted both in transit and at rest, ensuring that unauthorized access renders the data unreadable without a decryption key. Moreover, strict access controls have to be implemented to ensure that only authorized personnel can view or analyze genomic data. The adherence to established regulatory frameworks, such as HIPAA (Health Insurance Portability and Accountability Act) in the U.S. and GDPR (General Data Protection Regulation) in Europe, should be considered in order to mandate the secure handling, storage, and sharing of sensitive health information. All personal identifiers must be removed from datasets using techniques, such as data masking or aggregation, to prevent reidentification. Finally, establishing a data breach response plan can minimize harm by quickly identifying, containing, and notifying affected individuals in the event of a breach.

Another important issue to consider is the provision of culturally appropriate consent. Indeed, obtaining informed consent from diverse populations requires sensitivity to cultural, linguistic, and historical contexts. Many underserved communities have faced exploitation in research, making trust-building essential. Best practices should include the use of accessible language tailored to the literacy levels of participants to ensure clear communication, avoiding technical jargon that may confuse or mislead. The incorporation of culturally relevant examples and analogies may be useful to explain how genomic data will be used; the engagement of community leaders or liaisons may facilitate trust. The potential risks, benefits, and limitations of participating in genomic research should be clearly outlined. Finally, the adoption of dynamic consent models enables participants to update their consent preferences over time as new uses for their data emerge. As a practical example, the Navajo Nation has developed culturally informed genetic research policies through active engagement with community members, including workshops, meetings, and informational materials [80].

Last but not least, genomic medicine must be deployed with attention to sociocultural contexts, ensuring that underserved populations benefit equally from advances in precision medicine. Strategies for promoting equity include infrastructural, educational and political actions. As a matter of fact, financial resources have to be provided for building research infrastructure in low- and middle-income countries (LMICs). Culturally appropriate educational materials that explain genomic concepts in accessible ways should be realized. Cooperation with policymakers has to be established to reduce systemic barriers to accessing genomic tools, such as high costs or a lack of insurance coverage for genetic testing. Initiatives, like the Global Alliance for Genomics and Health (GA4GH), promote open-access databases that reflect global diversity while safeguarding privacy.

Recommendations for the implementation of these aspects include the following: (i) community engagement through the active involvement of underrepresented groups in the design and implementation of genomic studies (i.e., consulting with community leaders during study planning); (ii) capacity building, training researchers from underrepresented communities to lead genomic studies, and fostering local expertise; and (iii) global collaboration supporting initiatives like GA4GH that emphasize standardized pipelines, quality benchmarks, and responsible data-sharing practices.

By embedding these ethical principles into genomic medicine workflows, researchers can build trust, promote equity, and ensure that all populations benefit from the promise of precision medicine.

### 4.6. Global Collaboration and Data Sharing

International collaboration is essential for building inclusive, high-quality reference datasets and harmonizing imputation standards. Genomic research and clinical implementation benefit immensely from shared resources, standardized practices, and diverse data representation. Initiatives like the Global Alliance for Genomics and Health (GA4GH) play a pivotal role in fostering responsible data sharing while addressing privacy and equity concerns [81].

Key pillars for global collaboration include the following: (i) standardized pipelines and quality benchmarks; (ii) responsible data sharing with privacy safeguards; and (iii) open-access genomic databases reflecting global diversity. In particular, the adoption of harmonized workflows ensures consistent data processing, analysis, and reporting across institutions worldwide. This includes setting standards for variant calling, imputation accuracy, and quality control metrics. In this context, organizations, such as GA4GH, Genome in a Bottle (GIAB), and the International Genome Sample Resource (IGSR), provide benchmark datasets and tools to validate pipelines. Moreover, effective data sharing requires robust privacy frameworks to protect individuals while enabling scientific progress. These might include the following: (i) compliance with regulations, such as adherence to HIPAA, GDPR, and other regional privacy laws, ensuring the secure handling of genomic data; (ii) the encryption of genomic datasets both in transit and at rest to prevent unauthorized access; and (iii) tiered access models where sensitive data is only available to authorized researchers under strict governance policies. The GA4GH Framework for Responsible Sharing of Genomic Data provides actionable guidelines to balance privacy with accessibility (https://www.ga4gh.org/). Finally, open-access databases, like 1000 Genomes, gnomAD, and TOPMed, are critical for improving imputation accuracy across diverse populations. However, these databases must prioritize the equitable representation of underrepresented populations to avoid perpetuating disparities in research outcomes. In this context, The African Genome Variation Project has significantly improved representation of African populations in genomic studies.

Among the challenges in the genomic global collaboration of interest are the following: (i) the underrepresentation of populations, where many genomic datasets are biased toward individuals of European ancestry, limiting the generalizability of findings to other populations; (ii) data standardization barriers, since variations in regulatory requirements, technical infrastructure, and resource availability across countries hinder global standardization efforts; and (iii) ethical concerns, since ensuring that data sharing respects the rights of participants from historically underserved communities continues to be a major challenge.

Based on the above, we propose the following recommendations for strengthening global collaboration:

- Expand Diversity in Reference Panels: Actively recruit participants from underrepresented populations to create more inclusive datasets, and support initiatives like H3Africa that focus on increasing the genomic research capacity in Africa.
- Promote Capacity Building in Low- and Middle-Income Countries (LMICs): Provide financial support for infrastructure development, training programs, and research initiatives in LMICs. Collaborate with local institutions to develop culturally appropriate consent processes.
- Facilitate Open Data Access with Safeguards: Encourage open-access policies while implementing strong governance frameworks to protect participant privacy.
- Encourage Cross-Border Collaboration: Establish international consortia focused on specific diseases or traits (e.g., the COVID-19 Host Genetics Initiative). Share best practices through global workshops, conferences, and working groups.
- Leverage Technology for Secure Data Sharing: Use federated analysis models that allow researchers to analyze data locally without transferring it across borders. Blockchain technology could be explored as a tool for ensuring transparency and security in data-sharing agreements.

In summary, global collaboration is critical to advancing genomic medicine equitably and responsibly. Standardized pipelines, responsible data-sharing practices, and inclusive reference panels, and initiatives like GA4GH can help ensure that genomic research benefits all populations. However, these efforts must be matched with investments in infrastructure, ethical safeguards, and policies that prioritize equity.

## 5. Future Perspectives

As genome-wide association studies (GWASs) transition from research applications to clinical implementation, several strategic priorities must guide their evolution to ensure their safe, effective, and equitable integration into healthcare. First, improving the ancestral diversity of reference panels remains a critical need. Global sequencing initiatives should prioritize the inclusion of underrepresented populations, particularly from regions with high disease burdens and limited access to genomic technologies. Broader representation will enhance the imputation accuracy, strengthen polygenic risk models, and reduce the disparities in clinical genomic interpretation. Second, the development of hybrid genotyping approaches offers a promising pathway forward. By combining the direct sequencing of high-impact loci with the imputation of polygenic backgrounds, these methods balance cost, accuracy, and clinical utility. As sequencing costs decline, integrated pipelines may become standard, employing targeted sequencing for pharmacogenomic markers (e.g., *CYP2C19*) alongside broad-scale imputation for PRS construction. Third, the refinement of population-specific and ancestry-aware calibration methods for polygenic risk scores should continue. As PRS becomes increasingly embedded in screening and therapeutic decisions, calibration strategies will be essential in order to ensure validity across diverse populations. Fourth, regulatory frameworks must evolve to evaluate and govern the clinical use of genomic risk tools. In the United States, the Food and Drug Administration (FDA) provides oversight for diagnostics; in Europe, this role is fulfilled by the European Medicines Agency (EMA). As polygenic scores enter clinical pathways, clear criteria for analytical validity, clinical utility, and equity impact will be required. Finally, ethical and societal implications must remain central. The clinical use of imputed or probabilistic genomic data raises enduring questions about informed consent, data stewardship, the communication of uncertain results, and the potential to reinforce systemic inequities. Research into these areas should develop in parallel with technical innovation to ensure that the future of clinical genomics is transparent, trustworthy, and equitable.

## 6. Conclusions

Genome-wide association studies have ushered in a new era of precision medicine, enabling risk prediction, individualized treatment strategies, and novel insights into complex traits. However, the clinical translation of the GWAS findings relies heavily on the accuracy and interpretability of the underlying genomic data, particularly when imputation is used to infer untyped variants. This review has outlined the major technical, clinical, and ethical challenges associated with genotype imputation in clinical genomics. Key recommendations include prioritizing the direct genotyping of critical variants, validating imputation performance across ancestries, applying rigorous quality control, and ensuring the transparent reporting of variant-level confidence. We have also emphasized the importance of addressing ancestry-related biases through improved reference panels, local ancestry modeling, and recalibrated PRS frameworks. By implementing these best practices, researchers and clinicians can advance a more robust, reproducible, and inclusive use of GWASs in healthcare. The promise of genomic medicine is profound, but its equitable realization demands a sustained commitment to scientific rigor, transparency, and population diversity. We urge all stakeholders, researchers, clinicians, and policymakers, to prioritize these practices to ensure that the benefits of genomics are shared across all populations.

## Figures and Tables

**Table 1:** Summary of commonly used genotype imputation algorithms and their recommended use cases.

Algorithm	Strengths	Weaknesses	Optimal Context
IMPUTE2 [24]	High accuracy for common variants; extensively validated in population-based studies	Computationally intensive	Smaller datasets, studies requiring high accuracy for common variants
Beagle [25]	Fast, integrates phasing and imputation	Less accurate for rare variants	Large datasets, high-throughput studies
Minimac4 [26]	Scalable, optimized for low memory usage	Slight accuracy trade-off	Very large datasets, meta-analyses
GLIMPSE [27]	Effective for rare variant in admixed populations	Computationally intensive	Admixed cohorts; studies focused on rare variants
DeepImpute [28]	Captures complex patterns; potential for high accuracy	Requires large training datasets; less validated	Experimental settings with rich computational resources

**Table 2:** Summary of common imputation quality metrics and recommended thresholds.

Metric	Definition	Common Threshold	Interpretation
INFO Score	Squared correlation between imputed and true genotypes	>0.8 (typical)	High confidence; tool-specific
Imputation r^2^	Proportion of variance explained	>0.8	Regression-friendly; may differ by tool
Minor Allele Frequency (MAF)	Frequency of the less common allele	>0.01 (for inclusion)	Rare variants are less reliably imputed
Hard-Call Concordance	% match to gold-standard genotypes	>95%	Best for validating imputation accuracy directly

**Table 3:** Reported imputation accuracy by population, variant frequency, and reference panel (2015–2024).

Year & [Ref]	Population	Variant Frequency	Accuracy Metric	Imputation Method	Reference Panel	Sample Size	Accuracy
2023 [40]	European	Rare (MAF < 0.005)	r^2^	BEAGLE	PGP-UK Cohort	452,264	r^2^ = 0.43
2023 [40]	European	Common (MAF 0.2–0.5)	r^2^	BEAGLE	PGP-UK Cohort	452,264	r^2^ = 0.95
2024 [41]	British (GBR)	Rare (MAF < 10%)	r^2^	Not specified	GEL Panel	200,000	r^2^ = 0.60
2024 [41]	British (GBR)	Very Rare (MAF < 2%)	r^2^	Not specified	GEL Panel	200,000	r^2^ = 0.75
2024 [33]	Saudi Arabian	Rare (MAF 1–5%)	Mean Rsq	TOPMed	TOPMed Panel	1061	R^2^ = 0.79
2024 [33]	Vietnamese	Rare (MAF 1–5%)	Mean Rsq	TOPMed	TOPMed Panel	1264	R^2^ = 0.78
2024 [33]	Thai	Rare (MAF 1–5%)	Mean Rsq	TOPMed	TOPMed Panel	2435	R^2^= 0.76
2024 [33]	Papua New Guinean	Rare (MAF 1–5%)	Mean Rsq	TOPMed	TOPMed Panel	776	R^2^ = 0.62
2024 [42]	Japanese	Rare (MAF < 5%)	Aggregated r^2^	Minimac4	Japanese WGS-enhanced panel (1KG + 7K)	51,777	Improved over TOPMed
2023 [43]	Thai	Ultra-Rare (MAF < 0.001)	Genotype Concordance Rate (GCR)	GenomeAsia	GenomeAsia Panel	412	Median GCR = 0.97

**Table 4:** Validation metrics recommended to compare imputed genotype stratified by ancestry.

Metric	Definition
Concordance	Assess overall agreement between imputed and directly typed genotypes, calculating the proportion of matching calls across all variants and samples
Sensitivity (True Positive Rate)	Determine the proportion of true positives correctly identified by imputation, indicating the ability of imputation to detect variants that are present
Specificity (True Negative Rate)	Calculate the proportion of true negatives correctly identified by imputation, indicating the ability of imputation to correctly exclude variants that are absent
Positive Predictive Value (PPV)	Assess the proportion of imputed calls that are actually true positives, providing a measure of the reliability of positive predictions
Negative Predictive Value (NPV)	Determine the proportion of imputed non-calls that are actually true negatives, providing a measure of the reliability of negative predictions

**Table 5:** Best practices for transparent reporting of imputed genotypes in clinical genomic reports.

Variant	Genotype	Imputation Status	Quality Score	Interpretation
CYP2C19*2	*1/*2	Imputed	INFO = 0.85	Reduced function allele. Consider alternative therapy, especially in individuals of African ancestry
SLCO1B1*5	T/C	Imputed	r^2^ = 0.91	Increased statin-associated myopathy risk. May warrant dose adjustment or alternative statin use
*VKORC1*-1639G>A	A/A	Imputed	INFO = 0.87	Higher warfarin sensitivity. Consider lower starting dose per dosing guidelines
DPYD*2A	C/T	Imputed	INFO = 0.73	Partial DPD deficiency. Elevated risk of fluoropyrimidine toxicity. Consider alternative dosing
TPMT*3C	G/A	Imputed	r^2^ = 0.78	Decreased TPMT activity. Risk of thiopurine toxicity; consider dose reduction or alternative therapy

## Data Availability

The data are contained within the text.

## Abbreviations

The following abbreviations are used in this manuscript:

GWASs	Genome-Wide Association Studies
PRS	Polygenic Risk Score
SNP	Single-Nucleotide Polymorphism
LD	Linkage Disequilibrium
MAF	Minor Allele Frequency
PCA	Principal Component Analysis
OUD	Opioid Use Disorder
CNV	Copy Number Variation
CGH	Comparative Genomic Hybridization
WGS	Whole-Genome Sequencing
QC	Quality Control
HWE	1.3Hardy-Weinberg Equilibrium
MDS	Multidimensional Scaling
ML	Machine Learning
CNN	Convolutional Neural Networks
NGS	Next-Generation Sequencing
ddPCR	droplet digital PCR
HIPAA	Health Insurance Portability and Accountability
GDPR	Act General Data Protection Regulation
LMICs	low- and middle-income countries
FDA	Food and Drug Administration
EMA	the European Medicines Agency
